# Neuroimaging Biomarkers of Experimental Epileptogenesis and Refractory Epilepsy

**DOI:** 10.3390/ijms20010220

**Published:** 2019-01-08

**Authors:** Sandesh D. Reddy, Iyan Younus, Vidya Sridhar, Doodipala Samba Reddy

**Affiliations:** 1 Department of Neuroscience and Experimental Therapeutics, College of Medicine, Texas A&M University Health Science Center, Bryan, TX 77807, USA; 2 Texas A&M Institute for Preclinical Studies, College of Veterinary Medicine and Biomedical Science, Texas A&M University, College Station, TX 77843, USA

**Keywords:** epilepsy, epileptogenesis, seizures, biomarkers, imaging, MRI, PET, SPECT

## Abstract

This article provides an overview of neuroimaging biomarkers in experimental epileptogenesis and refractory epilepsy. Neuroimaging represents a gold standard and clinically translatable technique to identify neuropathological changes in epileptogenesis and longitudinally monitor its progression after a precipitating injury. Neuroimaging studies, along with molecular studies from animal models, have greatly improved our understanding of the neuropathology of epilepsy, such as the hallmark hippocampus sclerosis. Animal models are effective for differentiating the different stages of epileptogenesis. Neuroimaging in experimental epilepsy provides unique information about anatomic, functional, and metabolic alterations linked to epileptogenesis. Recently, several in vivo biomarkers for epileptogenesis have been investigated for characterizing neuronal loss, inflammation, blood-brain barrier alterations, changes in neurotransmitter density, neurovascular coupling, cerebral blood flow and volume, network connectivity, and metabolic activity in the brain. Magnetic resonance imaging (MRI) is a sensitive method for detecting structural and functional changes in the brain, especially to identify region-specific neuronal damage patterns in epilepsy. Positron emission tomography (PET) and single-photon emission computerized tomography are helpful to elucidate key functional alterations, especially in areas of brain metabolism and molecular patterns, and can help monitor pathology of epileptic disorders. Multimodal procedures such as PET-MRI integrated systems are desired for refractory epilepsy. Validated biomarkers are warranted for early identification of people at risk for epilepsy and monitoring of the progression of medical interventions.

## 1. Introduction

**Figure 1:**
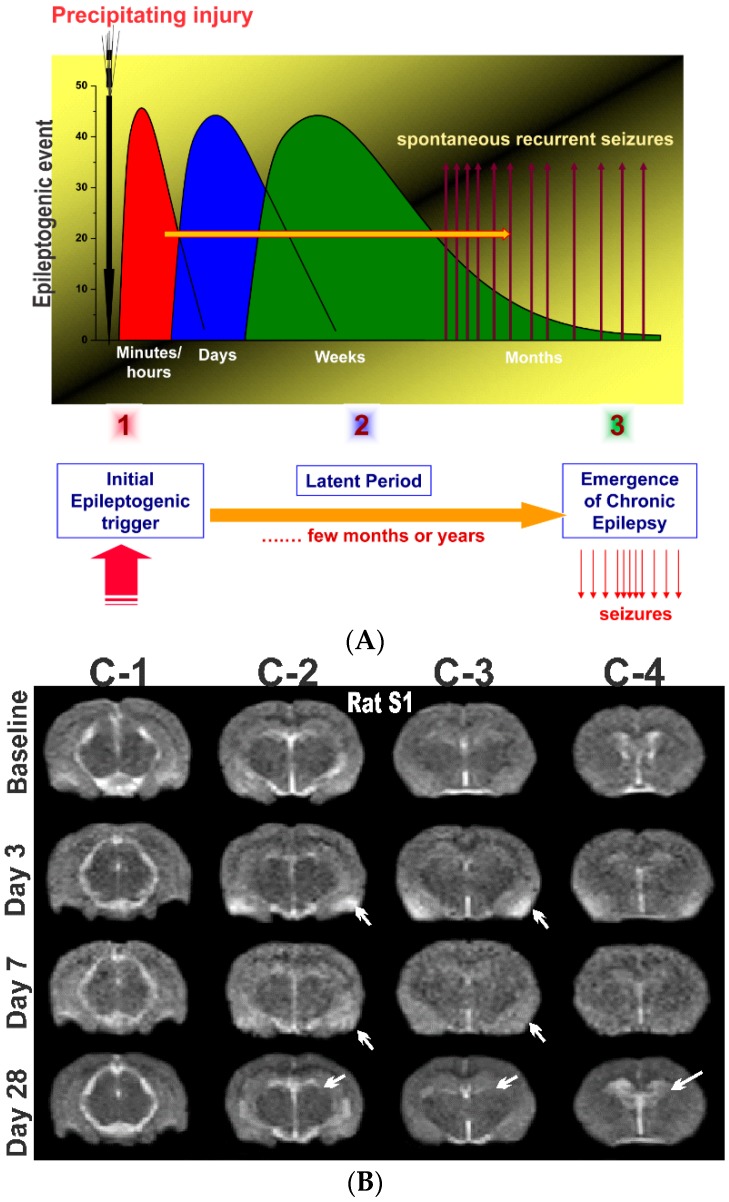
The process of epilepsy development and MRI biomarkers. (**A**) Epileptogenesis can be described in three progressive stages: (1) the initial injury (epileptogenic trigger); (2) the latent period (silent period with no seizures); and (3) chronic period with spontaneous recurrent seizures. The initial precipitating factor, such as brain injury, infections, stroke, and status epileptics, activates diverse signaling events, such as inflammation, oxidation, apoptosis, neurogenesis and synaptic plasticity, which eventually lead to structural and functional changes in neurons. These changes are eventually manifested as abnormal synchronized hyperexcitability and spontaneous seizures. (**B**) Representative MR images of brain before and after exposure to the organophosphate DFP in rats. T2-weighted coronal images showing the progressive changes in brain edema and damage at 3, 7 and 28 days post-DFP exposure. White arrows signify areas of pathological abnormalities. Overall, the hippocampus, limbic structures, and cortical regions show striking atrophy and lesions, while fluid expansion is evident in the lateral ventricles. C-1, C-2, C-3 and C-4 represents various coronal sections from rat brain. Animal use protocol was approved by the Institutional IACUC (#2017-0261) on 10/27/2017.

Epilepsy is the most complex brain disorder characterized by spontaneous recurrent seizures, which are abnormal electrical discharges in the brain. Epilepsy affects 1.2% of the U.S. population and about 40% exhibits intractable seizures that do not respond to antiepileptic drugs. However, there currently is no treatment available that prevents epilepsy following an epileptogenic insult by genetic or acquired conditions. Epileptogenesis is a complex process through which a normal brain is converted into a brain debilitated by recurrent seizure activity. This process ensues after several plastic changes occur in the brain [1,2]. Epileptogenesis evolves through three distinct phases: the initial injury phase, the latent phase, and the chronic phase (Figure 1A). An insult to the brain such as traumatic brain injury (TBI), infection, neurotoxin exposure, or stroke characterizes the initial injury phase. These insults activate a host of signaling cascades, triggering the epileptogenic pathway. The subsequent rearrangement of synaptic circuitry, neuronal damage, neurogenesis, and synchronized hyperexcitability characterize the latent phase. This phase has an unpredictable duration without any clinical manifestations, and is the primary opportunity for preventative intervention [3]. Latent changes eventually result in spontaneous seizure activity, marking the onset of the chronic phase.

There are over two dozen antiepileptic drugs (AEDs) for the treatment for epileptic seizures. However, approximately 40% of patients with epilepsy have intractable seizures that are pharmacoresistant to currently available AEDs [4,5]. Moreover, AEDs on the market provide symptomatic treatment and are only capable of controlling seizure occurrence but provide little impact on the underlying disease [6,7]. The current AEDs do not have antiepileptogenic effect, partly because the mechanisms behind anticonvulsant and antiepileptogenic activity are distinct in the various forms of acquired epilepsy in humans [8].

Curing epilepsy is identified as the top priority in the National Institutes of Health Benchmarks for Epilepsy Research. Many research efforts, therefore, are focused on investigating the pathophysiologic mechanisms underlying epileptogenesis. There are crucial gaps in knowledge of the neural network pathways that are responsible for the spatial and temporal events underlying the development of epilepsy [2]. To address these challenges, researchers are increasingly drawing upon neuroimaging modalities to obtain information about the plastic changes and alterations that occur in a brain during epileptogenesis [9,10,11].

Experimental neuroimaging systems are valuable to characterize early biomarkers in epilepsy. Animal models are also effective for characterizing the different stages of epileptogenesis, providing information about processes that cannot be studied in patients. Imaging biomarkers in preclinical models of epilepsy have tremendous value for non-invasive examination of structural, functional, and molecular changes in the brain. Several established neuroimaging modalities have aided in preclinical efforts for screening novel therapeutic interventions for epilepsy. Over the past decade, many biomarkers for epileptogenesis have been tested in characterizing neuronal cell loss (neurodegeneration), neuroinflammation, blood-brain barrier (BBB) alterations, changes in neurotransmitter density, neurovascular coupling, cerebral blood flow (CBF) and volume (CBV), network connectivity, and neuronal metabolic activity in the brain (Table 1).

Imaging of epileptogenic biomarkers with magnetic resonance (MR), positron emission tomography (PET), and single photon emission computer tomography (SPECT) has significantly contributed to our understanding of the pathophysiological mechanisms that underlie the development of epilepsy [12]. Preclinical animal models and clinical studies have produced evidence for several potential biomarkers for epileptogenesis. These studies are highlighted in the following sections and have been critical in establishing the specificity of imaging biomarkers associated with the different stages of epileptogenesis. These biomarkers are undoubtedly an important tool for predicting the seizure potential of tissue and longitudinally monitoring disease progression. Data collected from neuroimaging studies have allowed for accelerated screening of AEDs and contributed to the growing number of AEDs currently undergoing clinical trials. The translational nature of these studies has also greatly contributed to clinical advancements because the same imaging techniques used in animal models can be easily applied to patients. Therefore, in vivo imaging biomarkers represent a powerful tool to investigate the complex brain network dynamics that occur at different stages of epileptogenesis.

In this article, we describe various neuroimaging biomarkers in experimental epileptogenesis and refractory epilepsy. We highlight embracing these neuroimaging modalities as experimental biomarkers for in vivo monitoring of neuropathology and guide therapeutic response of interventions for modifying the development of epilepsy.

An epilepsy biomarker is defined as an objectively measured characteristic of a normal or pathologic epileptogenic process [13,14]. Identification and validation of biomarkers of epileptogenesis and ictogenesis might predict the development of an epilepsy, identify seizure generating regions, and evaluating therapeutic intervention for curing epilepsy [15,16]. The three main components of an epilepsy biomarker discovery consists of identification, validation and translation. Neuroimaging technologies, such as electroencephalogram (EEG), MRI and fMRI, provides in vivo tools in the identification of epilepsy biomarkers.

## 2. MRI and Subtypes in Preclinical Models of Epilepsy

MRI is a widely used in vivo imaging modality for detecting structural and functional changes in the brain. MRI is primarily used to measure microstructural and functional changes in the brain at the network level. MRI is also effective in assessing hemodynamics with high spatial resolution [17,18]. By generating magnetic fields, radio frequency pulses, and field gradients, an MRI scanner can generate radiological images of the brain and other organs of the body [18]. MRI is the imaging modality of choice for neurological cancers and many other diseases of the nervous system including epilepsy [17].

No radioactive material is required for MRI, making it a non-invasive imaging modality. The results from MRI have high translational value because the same imaging approaches can be used in both animal models and patients. In this report, we describe various MRI techniques and sequences, and their applications in various animal models of epilepsy. We provide a detailed review of the preclinical studies utilizing MRI imaging and their major findings. In the subsequent sections, the structural and functional alterations that can be imaged using MRI are summarized. These include pathophysiologic changes such as edema, microhemorrhages, CBF and CBV alterations, mossy fiber sprouting, BBB impairment, cell swelling, neurovascular coupling, and metabolic changes.

### 2.1. T1 and T2 Weighted MRI for Edema and Microhemorrhages

Anatomical imaging with MRI is most commonly achieved by unique relaxation times (T1 and T2) which characterize various tissues and pathologies. Anatomic imaging is the basic form of MRI that is part of almost all imaging modalities with this technique. Achieving T1 or T2 relaxation times can be done by adjusting timing parameters in MRI pulse sequence generation. T1-weighting is typically used in human imaging to assess anatomical detail, whereas T2-weighting is primarily used in looking at pathological lesions.

T1 and T2 relaxation times are largely based on the relaxation time of water in different tissue types. Tissue substances with the highest amount of water have longer relaxation time. For example, cerebral spinal fluid (CSF), which has a high water content, has a long relaxation time. Relaxation-based MRI contrast is particularly useful for imaging pathologic alterations such as edema, BBB impairment and microhemorrhage in animal models of epilepsy (Table 2).

In status epilepticus (SE) models, T1- and T2-weighted MRI relaxation times increased after the onset of SE and peaked at 24 h [21,27]. Once the edema was reabsorbed from the edematous lesion, normalization of the contrast was observed within 48–72 h. Furthermore, progressive atrophy of the hippocampus and cortex can be observed post-SE [19,20,21,22,23,24,25,26,27]. BBB breakdown is also seen on T2-weighted MRI in the thalamus within 2 h of SE [22].

In epileptogenic models of stroke and TBI, pathological changes such as acute edema, reabsorption, and atrophy can be observed in a time-course fashion [28]. Hemorrhage and microbleeds in stroke and TBI models can also be detected with T2-weighted MRI [29]. In the lateral fluid-percussion injury (LFPI) model of TBI, hippocampal and cortical atrophy is observed 3 h after injury with progression seen for up to 6 months [32].

As demonstrated by the studies summarized above, T2-weighted MRI represents a highly sensitive technique to characterize edema, microhemorrhages, lesion extent, and microstructural atrophy in animal models of epilepsy. However, there has been limited MRI study of neuronal injury progression in rodents, especially after organophosphate intoxication models [33]. We investigated short and long-term neuronal abnormalities in rats following exposure to the nerve agent soman and related organophosphates [34]. T2-weighted MR images were acquired from control and soman at 90-days post-exposure using a Siemens MRI scanner. Soman-exposed rats showed drastic hippocampal atrophy, indicating severe damage and neuronal loss. These animals displayed major increases in ventricle volumes and T2 times, signifying cerebrospinal fluid expansion in compensation for tissue atrophy. In rats exposed to the organophosphate diisopropylfluorophosphate (DFP), progressive increase in hippocampal and cortical damage was noted a few days after exposure (Figure 1), indicating severe lesions and neuronal injury. These rats displayed progressive increases in lateral ventricular volumes at 3, 7, and 28 days after DFP (Figure 1), indicating strong fluid expansion in obvious compensation for neuronal atrophy.

### 2.2. Contrast Agent Gadolinium for Cerebral Blood Flow, Volume and BBB Integrity

Contrast-enhanced MRI with gadolinium is utilized in epilepsy models to measure alterations in CBF, CBV, and BBB integrity (Table 3). CBF and CBV can be mapped using the dynamic contrast-enhanced approach similar to methods used in patients. Gadolinium can cause acute kidney injury in patients with renal dysfunction, although this does not limit its clinical translational potential.

In SE models, hemodynamic imaging shows increased CBF and CBV in the amygdala, characteristic of vascular reorganization following SE [40]. These findings demonstrate that epileptogenesis can be tracked by measuring associated hemodynamic changes.

Damage to the BBB resulting from epileptogenesis can be assessed on T1-weighted MRI after gadolinium injection. In kainic acid (KA)-induced SE, BBB damage in specific brain regions was reported for up to 6 weeks post-SE [35]. Similar methods have also been used to monitor the effect of rapamycin and isoflurane treatment on BBB impairment after SE [36,37].

The findings described above demonstrate the multiple applications of contrast-enhanced MRI in animal models of epilepsy. They also expand on the role of hemodynamic changes and BBB impairment as potential biomarkers for epilepsy. These studies also demonstrate the potential of contrast-enhanced MRI for further characterization of the latent phase of epileptogenesis.

### 2.3. Other Contrast Agents Such as Iron Oxide and Manganese

Other less commonly used contrast agents such as iron oxide and manganese have also been used in MRI animal epileptic studies. Iron oxides are considered negative contrast agents because their superparamagnetic property shortens T2 relaxation times and darkens affected tissue [41]. Studies with iron oxide contrast MRI have been conducted in pilocarpine-induced SE models. Iron-filled nanoparticles have been used to detect myeloid cells during chronic SE [42]. Another study used iron T2-weighted MRI to track transplanted iron-labeled bone marrow stem cells in pilocarpine-induced SE [43].

Manganese-enhanced MRI (MEMRI) is based upon the paramagnetic property of manganese (Mn^2+^), which shortens T1 relaxation times [44]. Manganese is considered a positive contrast agent because it brightens affected tissue in T1-weighted images [41]. Mn^2+^ has the same radius and charge as calcium (Ca^2+^) and thus acts as a Ca^2+^ analog in the brain, entering cells through Ca^2+^ channels and transporters and binding to Ca^2+^ binding sites [44]. Thus, through unique mechanisms of action, MEMRI, as well as iron oxide contrast MRI, have elucidated many different properties of epileptogenesis (Table 4).

Several factors influence the rate of Mn^2+^ accumulation in brain tissue including blood brain barrier (BBB) permeability. In functional MEMRI studies, Mn^2+^ permeability is increased by co-administration of mannitol. The MEMRI approach has been well-reported in animal models of epilepsy demonstrating mossy fiber sprouting and BBB leakage [47,48,49,50,51]. Mossy fiber sprouting can be observed with MEMRI scans in both KA and pilocarpine-induced SE.

A major drawback is that toxicity of manganese results in long-term structural and functional consequences. Furthermore, long-term exposure to manganese following systemic administration can lead to manganism, a neurodegenerative condition similar to Parkinson’s disease. This significantly limits its translation potential from animal models and restricts its clinical application [51]. Nonetheless, mossy fiber sprouting in epilepsy may represent an early biomarker of neuronal dysfunction in the latent phase of epileptogenesis.

### 2.4. Diffusion MRI for Cytotoxic Edema and Cell Swelling

Water diffusion is restricted by cellular membranes and myelin sheaths in a normal brain. Altered water diffusion is observed in pathologic processes that underlie epilepsy such as cytotoxic edema and cell swelling (Table 5).

Apparent diffusion coefficient (ADC) is the most commonly used parameter in diffusion MRI. Following brain insults that precede epileptogenesis, the ADC is reported to decrease in the first hours after insult. This initial and rapid diffusion drop down to about 60–80% of normal has been observed in both SE and focal lesion models of stroke and TBI [59]. Cytotoxic edema is characterized by the initial decline in diffusion and results from the inability to maintain a high extracellular and low intracellular sodium concentration. The osmolality gradient shifts water in to cells causing cell swelling. The initial decrease in ADC is followed by an increase in ADC that is specific for the resolution of cytotoxic edema. It was recently demonstrated that ADC changes in the hippocampus correlated with chronic hyperexcitability in both post-TBI and pilocarpine-induced models of epilepsy [54,59]. Furthermore, changes in ADC were only observed in rats that developed spontaneous limbic seizures as compared to those who did not in a hippocampal electrical stimulation model of epilepsy [60].

Diffusion tensor imaging (DTI) can be used to assess differences in white matter following chronic epilepsy [26]. DTI can also be used to assess microstructural changes during ictal and post-ictal states [61,62]. High-resolution DTI has also shown positive results in visualizing specific changes that occur in the hippocampus after various epileptogenic insults [57].

These studies demonstrate that cytotoxic edema, cell swelling, and microstructural changes all occur early in the epileptic brain, marking them as possible biomarkers in the development of epilepsy development. More studies will help elucidate whether these changes are specific to epileptogenesis or are secondary artifacts from the initial precipitating event.

### 2.5. Functional MRI for Neurovascular Coupling and Hemodynamic Activity

Functional MRI (fMRI) measures hemodynamic changes in the blood oxygen level in different parts of the brain by means of blood oxygen level-dependent (BOLD) sequences, thus attempting to indirectly measure neuronal activity [63]. fMRI with BOLD as well as diffusion tensor imaging has been applied to animal models of epilepsy to observe functional brain network connectivity and dysfunction (Table 6).

In LFPI models, fMRI revealed decreased connectivity between the ipsilateral and contralateral parietal cortex. A decrease in connectivity was also seen between the parietal cortex and hippocampus on the same side as the injury [65]. Furthermore, investigations of global network topology have demonstrated that epileptic brains exhibit altered functional brain networks when compared to control animals [64,68]. A recent study also demonstrated both EEG and resting state network connectivity changes after SE [70]. In amygdala kindling and electrical stimulation models, increased activity of subcortical structures and regions that depress cortical function was observed [68,69]. Additionally, seizure activity was observed to spread via multisynaptic connections through the amygdala [66]. Results from studies which have applied fMRI to animal models of epilepsy have clearly established its effectiveness for examining network connectivity changes post-SE. Whether any of the network connectivity alterations in limbic regions are specific biomarkers for epileptogenesis remains to be seen.

### 2.6. Magnetic Resonance Spectroscopy for Detecting Metabolites

Magnetic resonance spectroscopy (MRS) can be used to analyze different metabolites in the brain tissue. Proton (H^+^) MRS can be used for detection of high water and fat signal associated with epileptogenesis or epilepsy in several animal models (Table 7). Specifically, *N*-acetyl aspartate (NAA) can be detected in SE models as a marker of the chronic phase of the disease [71,72,73]. Furthermore, a recent study demonstrated that sodium selenite prevents changes in NAA in an SE model of epilepsy [74]. This study suggests a possible protective effect of sodium selenite. Furthermore, MRS showed a reduction of GABA-A receptor before the onset of seizures in pilocarpine-induced SE [75]. Studies using MRS have built on previous knowledge, but the role of other metabolites have yet to be investigated in epileptogenesis. This represents both a complex and novel area of research in epilepsy.

## 3. PET and SPECT Subtypes in Preclinical Models of Epilepsy

Nuclear imaging modalities including PET and SPECT are widely utilized in preclinical models of epilepsy. PET and SPECT are optimally used for functional and metabolic imaging using radiotracers by assessment of their biodistribution around the body. In the field of epilepsy, SPECT has been frequently applied for measuring CBF, while PET can elucidate neurotransmitter-receptor activity [82].

PET and SPECT imaging distinguish notable biomarkers in the processes underlying epileptogenesis. In the subsequent sections, the preclinical animal studies, which have elaborated on the application of tracers in PET and SPECT imaging, are summarized. Particularly, several radiolabeled tracers have been used in epilepsy research to visualize changes in brain metabolic activity, neurotransmitter receptor density, neuroinflammation, drug resistance, and BBB impairment (Table 1).

### 3.1. FDG for Imaging Brain Activation and Glucose Metabolism

Alterations in brain metabolic activity can be investigated by using the radiolabeled glucose analog, 2-fluoro-2-deoxy-d-glucose (FDG) in PET imaging. Alterations in glucose metabolism is an important feature underlying epilepsy and has several applications to neuronal death and dysfunction (Table 8).

In the KA and pilocarpine-induced models of SE, a sharp spike in brain glucose metabolism occurs following acute seizures [83]. Specifically, increased metabolic activity in the hippocampus, correlated with seizure severity, has been observed [89,90]. Reduced metabolic activity in several brain structures has also been reported around 3 days post-SE [73,84,85]. The reduced metabolic activity is thought to contribute to neuronal loss post-SE.

In the amygdala kindling model of epilepsy, seizure generation and propagation can be precisely timed. Observations from FDG-PET in the kindling model have demonstrated that several cortical and subcortical regions are recruited in the early stages of kindling [94].

The decreased metabolic activity observed in the early stages of epilepsy development has also been extensively investigated through FDG-PET. Decreased metabolic activity during early epilepsy correlated with duration of the latent phase and frequency of spontaneous seizures in the spontaneous recurrent seizure model of epilepsy [98]. In the LFPI model, FDG-PET parameters from the ipsilateral hippocampus were able to correctly predict the epileptic outcome in all LFPI cases [93]. Additionally, reduced metabolism in the hippocampus during the latent phase correlates with neuronal cell loss in rats [92].

Several studies have also investigated pharmacotherapeutic response in SE animals using FDG-PET parameters. A study showed that blocking serotonin did not prevent decreased metabolism and subsequent cell death post-SE [87,88]. Other studies have showed that short-term decreased metabolism can be prevented with fluoxetine treatment. A recent study determined that metyrapone treatment before SE is neuroprotective and prevents decreased metabolic activity post-SE [86,87,88]. In a genetic model of epilepsy in rats, acute vagus nerve stimulation was found to decrease hippocampal FDG uptake, correlating with increased duration of EEG spike wave duration [99,100].

Several studies summarized here have effectively applied FDG-PET imaging to study the major metabolic changes in epilepsy and assess how these changes contribute to neuronal death and dysfunction. From these studies, it is evident that decreased metabolic activity is a hallmark of early epileptogenesis. Additionally, recent studies have applied these techniques to demonstrate the neuroprotective effect of drugs that prevent alterations in metabolic activity. Drawing on the results of these studies validates the use of FDG-PET to study altered metabolic activity, in conjunction with seizure activity observed on EEG, as a potential biomarker of SE.

### 3.2. Molecular Imaging of Neurotransmitter Receptors

The balance between inhibitory and excitatory neurotransmission plays a critical role in epilepsy. Radiolabeled tracers have been developed to investigate neurotransmitter receptors and whether they are up- or down-regulated during epilepsy. The GABA-A receptor can be imaged with C or F-labeled flumazenil for PET and I-lonazenil for SPECT. Several studies used PET radioligands to investigate the density of GABA-A receptors in animal models of epilepsy (Table 9).

Decrease in GABA_A_ receptor density has been universally observed in all studies of KA and pilocarpine-induced SE. Decreases in GABA-A receptor density is observed in several hippocampus sublayers [101,103]. GABA-A receptor density is also reported to be decreased in cortical regions [104]. Additionally, a recent study proposed that decreased GABA-A receptor density characterizes the latent phase of epileptogenesis [101]. These results validate that down-regulation of GABA-A receptors on PET imaging during early epileptogenesis is a biomarker for epilepsy.

### 3.3. TSPO PET for Imaging of Brain Inflammation

Neuroinflammation is a pathological hallmark in the development of numerous neurological diseases including epilepsy [108]. Neuroinflammation can be characterized by targeting specific molecules expressed by immune-specific cells [109,110]. Translocator protein 18 kDA (TSPO) is a prominently-expressed protein which increases during brain inflammation. Several novel TSPO radioligands have been developed to quantify TSPO expression using PET [111]. Studies have thus used PET molecular imaging to investigate TSPO levels and neuroinflammation in epilepsy (Table 10).

All investigated studies have shown increased TSPO levels post-SE, peaking after 7–14 days and remaining elevated for up to 10 weeks [110,111,112,113,114]. Additionally, increased TSPO levels were observed specifically within in the limbic system 7 days post-SE [100]. In the SRS model of epilepsy, TSPO levels at 14 days post-SE were predictive of SRS frequency and severity of comorbidities during chronic SE [114]. In KA-induced SE, rats treated with isoflurane showed reduced TSPO levels within 5 days post-SE [37].

These studies have been critical in elucidating the role of neuroinflammation in epilepsy. These results highlight the application of TSPO-PET imaging as a non-invasive biomarker of neuroinflammation. Furthermore, these results warrant more investigation into the role of TSPO-PET in predicting seizure frequency and seizure severity in chronic SE.

### 3.4. Neuroreceptor Imaging in Epilepsy

Neuroinflammation PET has been widely used to study metabolic and neurotransmitter abnormalities in people with epilepsy. The development of several PET radioligands and their application in studying the neuroreceptor mechanisms of epilepsy is reviewed elsewhere [115]. Tracers binding to serotonin and GABA-A receptors have been used to identify the location of the epileptic focus. PET studies have examined the role of opioids, cannabinoids, acetylcholine, dopamine and drug transporter protein (P-glycoprotein) in seizure disorders and pharmacoresistant epilepsy [116]. Among the neuroreceptor system studied, an experimental model of frontal lobe nocturnal epilepsy linked to a β2 nicotinic receptor mutation has been investigated [117]. Autosomal dominant nocturnal frontal lobe epilepsy is a focal form of epilepsy characterized by seizures occurring during non-REM sleep. A recent study has shown changes in this cholinergic neuronal nicotinic receptor system in generalized epilepsy in humans [118]. It is suggested that changes in this system provide a reliable biomarker in idiopathic generalized epilepsy.

### 3.5. PET P-glycoprotein for Imaging of Drug Resistance

PET P-glycoprotein is another emerging imaging target that can be used to assess drug resistance in animal models of epilepsy. P-glycoprotein is an ATP-dependent efflux pump that is responsible for decreased drug accumulation. The activity of this pump leads to multidrug resistance in many cells [119]. Several P-glycoprotein inhibitors have been developed and investigated in animal models of epilepsy (Table 11).

Administration of the P-glycoprotein inhibitor tariquidar significantly alters the efflux rate constants of drugs such as quinidine and verapamil [121,122,125]. These studies indicate that P-glycoprotein activity may be altered in epilepsy. Additionally, tariquidar pre-treatment alters BBB clearance and efflux of injected tracer in epileptic rats [124].

These results suggest that PET imaging for drug resistance offers a sensitive biomarker for studying drug resistance in refractory epilepsy. These biomarkers also present interesting clinical application in aiding early diagnosis of refractory epilepsy and guiding early management of epilepsy in patients that do not respond to pharmacotherapy.

### 3.6. PET and SPECT for Imaging BBB Leakage

PET and SPECT imaging (much like MRI) have also proven effective in imaging BBB impairment in animal models of epilepsy. One study demonstrated that PET, SPECT, and MRI are all equally capable of sensitively detecting BBB disturbances in epileptogenesis. The study found that increased BBB permeability occurs 48 h post-SE predominantly in brain regions associated with epileptogenesis such as the hippocampus, piriform cortex, thalamus, and amygdala [124]. These results build on previous reports of BBB permeability as a possible biomarker for epileptogenesis and related neuronal excitability conditions [9].

## 4. Conclusions and Perspectives

**Figure 2:**
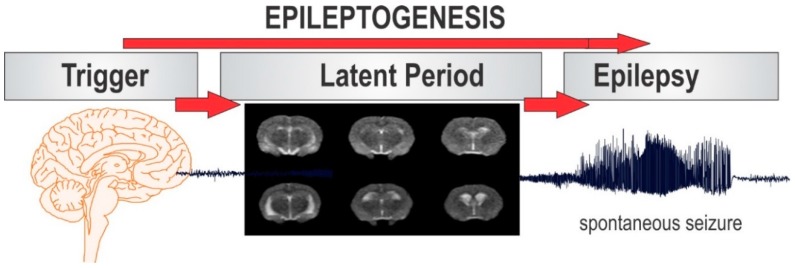
The cellular and molecular abnormalities that are observed during the latent period as predictive biomarkers of epileptogenesis. Various modalities as putative biomarkers of epileptogenesis include biochemical, neuroimaging, electrophysiological markers. Neuroimaging biomarkers can be developed based on cellular and mechanistic changes, such as neurodegeneration, astrocyte activation, microglial activation, vascular remodeling, axonal sprouting, oxidative stress and calcium deposition.

Epilepsy is a challenging disease to study because patients diagnosed with epilepsy present symptoms long after the pathologic process of epileptogenesis has ensued (Figure 2). In vivo imaging biomarkers represent a non-invasive and clinically translatable approach to identify early indications of epileptogenesis and longitudinally monitor disease progression. The imaging modalities discussed in this review, in combination with results from animal models of epilepsy, have significantly contributed to our understanding of the pathophysiologic mechanisms underlying epilepsy. However, novel applications of MRI, PET, and SPECT imaging in epilepsy models require key flaws to be addressed before they can fulfill their potential. Most significantly, few studies have investigated whether the alterations observed in various imaging models are indeed associated with epileptogenesis or just artifacts of the initial insult. This lack of specificity for the epileptogenic process is a common weakness of current studies evaluating biomarkers for epileptogenesis.

Nonetheless, in vivo imaging studies can be a reliable tool in various epilepsy research designs, especially for evaluating the effectiveness of AEDs in preclinical models. These imaging studies are valuable because of their high translational potential. Due to the non-invasive nature of these techniques, human testing faces fewer ethical and organizational obstacles. The broad spectrum of imaging modalities discussed in this review such as MRI, PET, and SPECT all represent highly specific and precise approaches for assessing and quantifying a host of underlying pathological changes in epileptogenesis. The ideal standard for determining in vivo imaging biomarkers is to combine imaging data with video-EEG monitoring. This concurrent analysis provides better correlation between the behavioral patterns of epileptogenesis and the anatomical and functional alterations observed through in vivo imaging.

Although MRI is a sensitive method for obtaining information about structural and functional changes in the brain, future development of microstructural contrasts can improve specificity [115]. This is necessary to reconstruct more detailed and complex information about the orientation of microstructures in the brain. PET and SPECT technology is also rapidly advancing with the advent of new radiotracers. These novel radiotracers will aid in expanding the range of molecular targets that can be studied. Drawing on PET ligands that are already applied in different species or in other diseases may also expand our ability to image vast alterations in epilepsy. Another exciting prospect lies in simultaneous multimodal imaging with both PET and MRI techniques, particularly in the small animal model. Application of multimodal imaging may allow for more precise analysis of structural, functional, and molecular changes in epileptogenesis. Beyond the detection of epileptogenic lesions on structural MRI and focal hypometabolism on PET, EEG-based Electric Source Imaging (ESI) and simultaneous EEG and functional MRI are applied for mapping epileptic activity. Recently, such an innovation was reported with clinical utility of PET-MRI integrated systems in epilepsy [126]. This quadrimodal imaging procedure was performed in a single session.

Overall, considering the latent nature of epileptogenesis, reliable, non-invasive, and clinically-translational models of epilepsy are necessary to study the pathophysiological changes throughout the process. While most studies discussed in this review used common animal models of epilepsy such as pilocarpine or KA-induced SE, kindling-induced seizures, or LFPI model of TBI, in vivo imaging techniques can be applied to a host of other relevant epilepsy models. There are many areas of need for biomarkers including identification of people at risk for epilepsy and reversing its progression with therapeutic interventions. A pressing area of need for biomarkers is to identify patients with refractory or pharmacoresistant seizures. Further discoveries in imaging biomarkers will undoubtedly allow for better characterization of the epileptogenic process and offer valuable insight into preventing or reversing the course of epilepsy.

## Figures and Tables

**Table 1:** Overview of in vivo imaging biomarkers for epilepsy.

Imaging Modality	Epilepsy Models	Potential Biomarker
T1, T2-weighted MRI	Post-SE, kindling, LFPI-TBI	T2-weighted signal hyperintensity for edema, gliosis, cell loss, BBB impairment
Contrast-enhanced MRI	Post-SE	Gadolinium, iron oxide, and magnesium enhanced signal change for mossy fiber, BBB breakdown, CBV and CBF changes
Diffusion MRI	Post-SE, kindling, LFPI-TBI	Changes in FA, perfusion, and diffusion for edema, axonal injury, and connectivity changes
Functional MRI	Post-SE, kindling, LFPI-TBI	Changes in BOLD signal for alterations in brain network connectivity and activity
MRS	Post-SE, kindling	Changes in NAA, mIns, GABA-A, glutamate, and glutamine, and glutathione for neuronal death and dysfunction
PET-FDG	Post-SE, kindling, LFPI-TBI, SRS	Changes in glucose metabolism for brain activation, metabolic alterations, and neuronal loss
PET-TSPO	Post-SE, SRS	Changes in TSPO for neuroinflammation
PET Radiotracers	Post-SE, kindling, SRS	PET radiotracers for neurotransmitter density, drug resistance, and BBB integrity

*Abbreviations*: SE, status epilepticus; TBI, traumatic brain injury; LFPI, lateral fluid-percussion injury; FA, fatty acid; SRS, spontaneous recurrent seizures; FDG, fluorodeoxyglucose; TSPO, 18-kDa translocator protein.

**Table 2:** List of T1 and T2-weighted magnetic resonance imaging (MRI)-based studies and potential biomarkers for epilepsy.

Epilepsy Model	Major Finding	Reference
SE-KA	Progressive atrophy of hippocampus within weeks	[19]
SE-pilo	CBV increase in the hippocampus and subcortical structures; Marked edema in areas corresponding to the highest T2-weighted intensity	[20]
SE-pilo	T2 relaxation time increased after 24 h and resolved within 48–72 h in rats that developed epilepsy	[21]
SE-pilo	Blood-brain barrier breakdown could be observed only in the thalamus after 2 h and disappeared by 6 h; Edema in the amygdala and cortex that disappeared progressively over a 5-day period	[22]
SE-pilo	T2 in the amygdala 30 days after SE had a strong correlation with hyperactivity in the novel open field.	[23]
SE-electrically	Progressive atrophy and thinning of hippocampus and cortex within weeks	[24]
MTLE-KA	T2 relaxation time correlated with number and duration of hippocampal paroxysmal discharges	[25]
Kindling	Brain structural differences between seizure prone and seizure resistance rats	[26]
4-AP	T2 relaxation times showed changes throughout the cerebral cortex, hippocampus, amygdala and medial thalamus, with complete recovery after 3 days	[27]
LFPI-TBI	Hippocampal and cortical atrophy starts at 3 h post-injury and continue to progress for up to 6 months	[28]
SE-pilo	Increased acute CBF to the parietal cortex and thalamus, but decreased CBF to the hippocampus	[29]
FSE	Reduced amygdala T2 relaxation times in high-magnetic-field MRI hours after FSE predicted experimental TLE	[30]
FSE	T2 relaxation times in hippocampus and amygdala 24 h after FSE correlated with spatial cognitive deficits	[31]

*Abbreviations*: SE, status epilepticus; KA, kainic acid; 4-AP, 4-aminopyridine; MTLE, medial temporal lobe epilepsy; FSE: febrile status epilepticus; TLE, temporal lobe epilepsy.

**Table 3:** List of gadolinium-based contrast MRI studies and potential biomarkers for epilepsy.

Epilepsy Model	Major Finding	Reference
SE-KA	BBB leakage at 1 day and 6 weeks after SE in the hippocampus, entorhinal cortex, amygdala and piriform cortex	[35]
SE-KA	Reduced BBB leakage during the chronic phase could contribute to the decreased seizure frequency in post-SE rats treated with rapamycin	[36]
SE-KA	Isoflurane prevented BBB dysfunction and neurodegeneration 48 h after SE	[37]
SE-pilo	Increased BBB permeability 48 h post SE in the hippocampus, piriform cortex, thalamus, and amygdala	[38]
LFPI-TBI	Acute BBB disruption in the cortex; BBB disruption 72 h-post correlated with seizure susceptibility	[39]

**Table 4:** List of iron oxide contrast and manganese-enhanced MRI studies and potential biomarkers for epilepsy.

Epilepsy Model	Major Finding	Reference
SE-pilo	Increased CBF and CBV associated with increased vessel density in amygdala post-SE	[45]
SE-pilo	Iron-filled nanoparticles used to detect myeloid cells during chronic phase SE	[42]
SE-pilo	Iron T2-weighted MRI was used to track transplanted iron-labeled bone marrow stem cells in SE	[43]
SE-KA	T1-weighted hyperintensity correlated with mossy fiber sprouting	[46]
SE-KA	Decrease in hypersensitivity during chronic phase	[47]
SE-KA	Lesions in hippocampus CA3 and CA1 subfields	[48]
SE-KA	T1-weighted hyperintensity correlated with axonal sprouting but not seizure activity	[49]
SE-KA	T1-weighted hyperintensity inversely correlated with frequency of spontaneous seizure	[50]
SE-pilo	T1, T2-weighted hyperintensity was not increased acutely post pilo-induced SE	[51]

**Table 5:** List of diffusion MRI studies and potential biomarkers for epilepsy.

Epilepsy Model	Major Finding	Reference
SE-KA, pilo	Increase in fractional anisotropy in the dentate gyrus several months post-SE associated with mossy fiber sprouting and axonal reorganization	[52]
SE-KA, pilo	Longitudinal changes in hippocampal diffusion due to astrocyte processes	[53]
SE-pilo	Diffusion changes correlate with epilepsy severity in mice	[54]
SE-pilo	Axonal plasticity and reorganization	[55]
SE-pilo	Drop in ADC hour after induced-SE correlated with long-term neuronal cell loss; diazepam reduced ADC drop	[56]
SE-pilo, LFPI	Layer-specific changed in hippocampus of rats	[57]
SE-electrically	Decreased diffusion normalized 9 days post-SE	[24]
SE-bicuculline	Rapid diffusion decrease up to 1 day post-SE	[58]
LFPI-TBI	Hippocampus changes correlated with chronic hyperexcitability	[59]
SS-electrically	Drop in hippocampal ADC and rise in cortical ADC in acute period; Elevated hippocampal and cortical ADC in latent/chronic phases	[60]
SRS	Microstructural changes and hypoperfusion in hippocampus and parietal cortex during ictal periods in cats	[61]
SRS	Decrease in hippocampus perfusion during postictal state compared to interictal state in cats	[62]
Kindling	Chronic white matter changes in seizure prone rats post-kindling	[26]

**Table 6:** List of functional MRI (fMRI) studies and potential biomarkers for epilepsy.

Epilepsy Model	Major Finding	Reference
SE-KA	Functional brain network disruption in chronic SE	[64]
SE-KA	Feasibility study for longitudinal studies combining EEG and fMRI	[64]
LFPI	Decreased connectivity after 4 months post-LFPI	[65]
Amygdala kindling	Seizure activity spread through multisynaptic connections to the amygdala in rhesus monkeys	[66]
Electrical stimulation	Increased activity of subcortical structures during impaired consciousness in rats	[67]
Electrical stimulation	Increased activity in regions that depress cortical function	[68]
Facial seizures	Reduced network connectivity in rats with white matter changes	[69]

**Table 7:** List of MR spectroscopy (MRS) studies and potential biomarkers for epilepsy.

Epilepsy Model	Major Finding	Reference
SE-KA	Reported metabolic and pathologic changes during disease progression post-SE	[71]
SE-KA	Reduced NAA levels and increased mIns, glutamine, and T2 relaxation time post-SE	[76]
SE-KA	Different metabolic parameters in dentate gyrus between two different clusters of rats 3 days post-SE	[77]
SE-pilo	Reported hypoxia, excitotoxicity, and neuronal damage post-SE	[78]
SE-pilo	Reduced NAA in the hippocampus post-SE	[79]
SE-pilo	Reduced NAA 2 days post-SE and negative correlation between glutathione and mIns with neurodegeneration in hippocampus	[72]
SE-pilo	Reduced NAA and Cr levels in the hippocampus and basal ganglia both acute and chronic	[73]
SE-pilo	Reduction of GABA-A and glutamate before onset of seizures	[75]
SE-pilo	Increased mIns in rats post-SE	[80]
Pilocarpine	Altered astrocytic and neuronal metabolism with dose-dependent reduction in glycogen in mice	[81]
SE-KA and amygdala kindling	Sodium selenate prevents changes in mIns and NAA levels, volumetric changes, and FA	[74]

**Table 8:** List of PET-FDG studies for brain glucose metabolism and potential biomarkers for epilepsy.

Epilepsy Model	Major Finding	Reference
SE-KA	Increased metabolic activity following limbic seizures	[83]
SE-KA, SRS	Reduced metabolism in several brain structures post-SE and at the onset of SRS	[84]
SE-pilo	Reduced metabolism post-SE	[73]
SE-pilo	Reduced metabolic activity 3 days post-SE	[85]
SE-pilo	Fluoxetine treatment prevented short-term decreased metabolism	[86]
SE-pilo	Blocking serotonin did not prevent decreased metabolism post-SE	[87]
SE-pilo	Metyrapone treatment before SE was neuroprotective and prevented decreased metabolism	[88]
SE-pilo	Increased metabolic activity in the hippocampus; Increased glucose uptake correlated with seizure severity	[89]
SE-pilo	Correlation between metabolic activity and seizure induction	[90]
SE-pilo	Decreased metabolic activity and brain connectivity	[91]
SE-pilo	Reduced metabolism in the hippocampus during the latent phase correlates with neuronal cell loss in rats	[92]
LFPI-TBI	Reduced metabolism predicted epilepsy outcome	[93]
Amygdala kindling	Cortical and subcortical regions metabolically active in the first stages of kindling	[94]
Amygdala kindling	Different patterns of time-depended perfusion in rhesus monkeys	[95]
PTZ kindling	Different glucose uptake between animals resistant vs non-resistant to kindling	[96]
Kindling	Low-frequency stimulation prevented decreased metabolism in the limbic system	[97]
SRS	Decreased metabolism during early epilepsy correlated with duration of latent phases and frequency of SRS	[98]
GAERS	Acute vagus nerve stimulation decreased hippocampus FDG uptake	[99]

*Abbreviations*: PTZ, pentylenetetrazol; GAERS, genetic absence epilepsy rats from Strasbourg.

**Table 9:** List of PET molecular imaging studies of neurotransmitter receptors and potential biomarkers for epilepsy.

Epilepsy Model	Major Finding	Reference
SE-KA	Decrease in GABA-A receptor density in several hippocampus sublayers	[101]
SE-KA	Decrease in GABA-A receptor density during latent phase	[102]
SE-KA	Decrease in GABA-A receptor density and affinity in the hippocampus in chronic SE	[103]
SE-pilo	Decrease in D_2_/D_3_ dopamine receptors in chronic SE	[104]
SE-pilo	Decrease in global mGluR5 metabotropic glutamate receptor; Focal decrease in amygdala and hippocampus during chronically	[105]
Kindling	Decrease in GABA-A receptor density	[106]
Cortical dysplasia	Decrease in GABA-A receptor density in several cortical regions	[107]

**Table 10:** List of PET molecular imaging studies for brain inflammation and potential biomarkers for epilepsy.

Epilepsy Model	Major Finding	Reference
SE-KA	Increased translocator protein (TSPO) levels in the limbic system 7 days post-SE	[100]
SE-KA	Reduced TSPO levels in rats exposed to isoflurane 5 days post-SE	[37]
SE-KA	Increased TSPO levels peak at 14 days post-SE	[110]
SE-pilo	Increased TSPO levels 7–14 days post-SE	[96]
SE-pilo	Increased TSPO levels 6 days post-SE	[112]
SE	Increased TSPO levels up to 10 weeks post-SE	[113]
SRS	TSPO levels 14 days post-SE predict SRS frequency and severity of comorbidities during chronic SE	[114]

**Table 11:** List of PET molecular imaging studies for drug resistance and BBB integrity and potential biomarkers for epilepsy.

Epilepsy Model	Major Finding	Reference
SE-KA	Tariquidar treatment increased tracer uptake, however P-glycoprotein expression and functionality did not differ between controls and epileptic rats	[120]
SE-pilo	Tariquidar pre-treatment enhanced differences in kinetic influx/efflux rate constant between controls and epileptic rats	[121]
SE-pilo	Effects of tariquidar and SE on 11C-Verapamil transport across BBB	[122]
Self-sustained SE	Increased TSPO levels in drug-resistant epileptic rats	[123]
SRS	Tariquidar pre-treatment altered tracer blood-brain clearance and efflux rate constant in non-responder epileptic rats	[124]
SRS	Tariquidar pre-treatment showed slight differences with 11C-quinidine between drug resistant and drug-sensitive rats	[125]
SE-pilo	Increased BBB permeability 48 h post-SE in the hippocampus, piriform cortex, thalamus, and amygdala	[38]

## Abbreviations

TBI	traumatic brain injury
AED	antiepileptic drug
BBB	blood brain barrier
CBF	cerebral blood flow
CBV	cerebral blood volume
MRI	magnetic resonance imaging
PET	positron emission tomography
SPECT	single photon emission computer tomography
CSF	cerebral spinal fluid
LFPI	lateral fluid percussion injury
MEMRI	manganese-enhanced MRI
ADC	apparent diffusion coefficient
DTI	diffusion tensor imaging
FA	fractional anisotropy
fMRI	functional MRI
BOLD	blood oxygen level-dependent
ASL	arterial spin labeling
SE	status epilepticus
MRS	magnetic resonance spectroscopy
NAA	*N*-acetyl aspartate
GABA	γ-aminobutyric acid
FDG	2-fluoro-2-deoxy-d-glucose
KA	kainic acid
SRS	spontaneous recurrent seizures
TSPO	Translocator protein 18 kDA PTZ, pentylenetetrazol
GAERS	genetic absence epilepsy rats from Strasbourg
mIns	myo-inositol
MTLE	medial temporal lobe epilepsy
4-AP	4-aminopyridine
FSE	febrile status epilepticus
